# 5-Chloro-1-nonyl-1*H*-benzimidazol-2(3*H*)-one

**DOI:** 10.1107/S1600536811047829

**Published:** 2011-11-16

**Authors:** Youssef Kandri Rodi, Fouad Ouazzani Chahdi, El Mokhtar Essassi, Santiago V. Luis, Michael Bolte, Lahcen El Ammari

**Affiliations:** a Laboratoire de Chimie Organique Appliquée, Université Sidi Mohamed Ben Abdallah, Faculté des Sciences et Techniques, Route d’immouzzer, BP 2202, Fès, Morocco; b Laboratoire de Chimie Organique Hétérocyclique URAC21, Faculté des Sciences, Université Mohammed V-Agdal, Av. Ibn Battouta, BP 1014, Rabat, Morocco; c Departamento de Quimica Inorganica & Organica, ESTCE, Universitat Jaume I, E-12080 Castellon, Spain; d Institut für Anorganische Chemie, J.W. Goethe-Universität Frankfurt, Max-von-Laue-Strasse 7, 60438 Frankfurt/Main, Germany; e Laboratoire de Chimie du Solide Appliquée, Faculté des Sciences, Université Mohammed V-Agdal, Avenue Ibn Battouta, BP 1014, Rabat, Morocco

## Abstract

The asymmetric unit of the title compound, C_16_H_23_ClN_2_O, comtains two independent mol­ecules in which the fused-ring systems are essentially planar, the largest deviation from the mean plane of each mol­ecule being 0.011 (2) Å and 0.016 (2) Å. The benzimidazole rings of the two mol­ecules make a dihedral angle of 66.65 (7)°. The nonyl substituents are almost perpendicular to the benzimidazole planes [C—N—C—C tosrsion angles = 96.0 (3) and 81.0 (2)°]. In the crystal, each independent molecule forms an inversion dimer *via* a pair of N—H⋯O hydrogen bonds. In one of the independent molecules, the terminal –CH_2_–CH_3_ group of the alkyl chain is disordered over two sets of sites with a refined occupancy ratio of 0.746 (7):0.254 (7).

## Related literature

For the pharmacological, biochemical and structural properties of benzimidazolo­nes, see: Al Muhaimeed (1997 ▶); Nakano *et al.* (2000 ▶); Scott *et al.* (2002 ▶); Zarrinmayeh *et al.* (1998 ▶); Zhu *et al.* (2000 ▶); Ouzidan *et al.* (2011*a* ▶,*b* ▶).
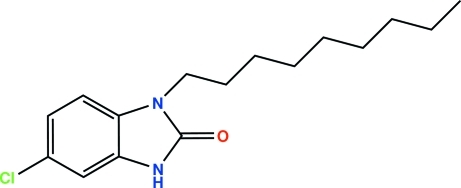


## Experimental

### 

#### Crystal data

C_16_H_23_ClN_2_O
*M* *_r_* = 294.81
Triclinic, 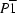

*a* = 5.51441 (17) Å
*b* = 15.6507 (4) Å
*c* = 20.0540 (6) Å
α = 71.807 (3)°
β = 86.612 (2)°
γ = 80.709 (2)°
*V* = 1622.59 (8) Å^3^
*Z* = 4
Cu *K*α radiation
μ = 2.06 mm^−1^
*T* = 296 K
0.43 × 0.20 × 0.16 mm

#### Data collection

Agilent SuperNova Dual (Cu at zero) Atlas diffractometer
Absorption correction: multi-scan (*SADABS*; Sheldrick, 1996 ▶) *T*_min_ = 0.640, *T*_max_ = 0.720
31859 measured reflections
6416 independent reflections
5705 reflections with *I* > 2σ(*I*)
*R*_int_ = 0.031

#### Refinement

*R*[*F* ^2^ > 2σ(*F* ^2^)] = 0.044
*wR*(*F* ^2^) = 0.124
*S* = 1.03
6416 reflections
379 parameters
9 restraints
H atoms treated by a mixture of independent and constrained refinement
Δρ_max_ = 0.46 e Å^−3^
Δρ_min_ = −0.32 e Å^−3^

### 

Data collection: *CrysAlis PRO* (Agilent, 2011 ▶); cell refinement: *CrysAlis PRO*; data reduction: *CrysAlis PRO*; program(s) used to solve structure: *SHELXS97* (Sheldrick, 2008 ▶); program(s) used to refine structure: *SHELXL97* (Sheldrick, 2008 ▶); molecular graphics: *XP* (Sheldrick, 2008 ▶); software used to prepare material for publication: *SHELXL97*.

## Supplementary Material

Crystal structure: contains datablock(s) I, global. DOI: 10.1107/S1600536811047829/im2336sup1.cif

Structure factors: contains datablock(s) I. DOI: 10.1107/S1600536811047829/im2336Isup2.hkl

Supplementary material file. DOI: 10.1107/S1600536811047829/im2336Isup3.cml

Additional supplementary materials:  crystallographic information; 3D view; checkCIF report

## Figures and Tables

**Table 1:** Hydrogen-bond geometry (Å, °)

*D*—H⋯*A*	*D*—H	H⋯*A*	*D*⋯*A*	*D*—H⋯*A*
N2—H2⋯O1^i^	0.83 (2)	1.96 (2)	2.778 (2)	170 (2)
N2*A*—H2*A*⋯O1*A*^ii^	0.85 (2)	1.95 (2)	2.7937 (18)	171.1 (19)

Symmetry codes: (i) 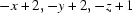; (ii) 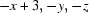.

**Fig. 1.**
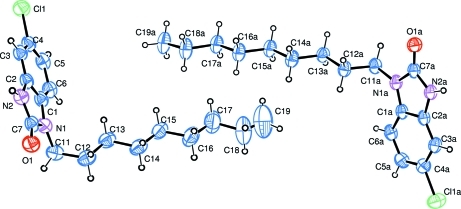
Molecular structure of the title compound. Displacement ellipsoids are drawn at the 50% probability level. H atoms are represented as small circles.

**Fig. 2.**
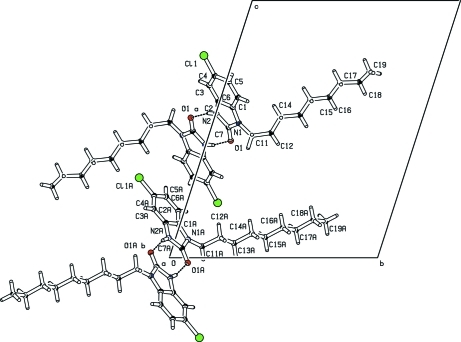
Centrosymmetric dimer of one of the independent molecules linked by hydrogen bonds.

## supplementary crystallographic information

### Comment

Benzimidazoles and their derivatives exhibit a number of important pharmacological properties, such as antihistaminic (Al Muhaimeed, 1997) anti-ulcerative (Scott *et al.*, 2002) and antiallergic (Nakano *et al.*, 2000). In addition, benzimidazole derivatives are effective against the human cytomegalovirus (HCMV) (Zhu *et al.*, 2000) and are also efficient selective neuropeptide Y Y1 receptor antagonists (Zarrinmayeh *et al.*, 1998).

As a continuation of our research work devoted to the development of substituted benzimidazol-2-one derivatives (Ouzidan *et al.*, 2011*a*, 2011*b*), we report the synthesis of a new benzimidazol-2-one derivative by action of nonyl bromide with 5-chloro-1,3-dihydrobenzimidazol-2-one. The reaction provided the title compound (Scheme 1).

The asymmetric unit of the title compound, C_16_H_23_ClN_2_O is built up from two independent molecules with different orientations as shown in Fig.1. The two fused five and six-membered rings building each molecule are almost planar with the maximum deviation of 0.011 (2) Å and 0.016 (2) Å for N2 in the first molecule (C1 to C19) and for C1A in the second molecule (C1A to C19A), respectively. The dihedral angle between the two benzimidazole rings is 66.65 (7)°. The nonyl groups are almost perpendicular to the benzimidazole planes as indicated by the torsion angles of C1—N1—C11—C12 = 96.0 (3) ° and C1A—N1A—C11A—C12A = 81.0 (2) °.

In the crystal structure, each molecule forms a hydrogen bonded centrosymmetrical dimer as shown in Fig.2.

### Experimental

To 5-chloro-1,3-dihydrobenzimidazol-2-one (0.2 g, 1.18 mmol), potassium carbonate (0.33 g, 2.38 mmol), and tetra-n-butylammonium bromide (0.04 g, 0.11 mmol) in DMF (15 ml) was added nonyl bromide (0.34 ml, 1.78 mmol). Stirring was continued at room temperature for 6 h. The salts were removed by filtration and the filtrate concentrated under reduced pressure. The residue was separated by chromatography on a column of silica gel with ethyl acetate/hexane (1/2) as eluent (Yield: 34%). Single crystals were isolated when the solvent was allowed to evaporate at room temperature.

### Refinement

The nonyl group of the first molecule shows a disordered –CH_2_—CH_3_ terminus as shown in the high values of the atomic displacement parameters. This group is refined with C18–C19 distance restraints to 1.510 Å. H atoms were located in a difference map and treated as riding with N—H = 0.86 Å, C—H = 0.93 Å (aromatic), C—H = 0.97 Å (methylene) and C—H = 0.96 Å (methyl) with *U*_iso_(H) = 1.2 *U*_eq_(aromatic, methylene) and *U*_iso_(H) = 1.5 *U*_eq_(methyl). The nitrogen bonded H atom was refined without fixed thermal parameters.

### Crystal data

**Table:**

C_16_H_23_ClN_2_O	*Z* = 4
*M**_r_* = 294.81	*F*(000) = 632
Triclinic, *P*1	*D*_x_ = 1.207 Mg m^−^^3^
Hall symbol: -P 1	Cu *K*α radiation, λ = 1.54184 Å
*a* = 5.51441 (17) Å	Cell parameters from 5000 reflections
*b* = 15.6507 (4) Å	θ = 5–50°
*c* = 20.0540 (6) Å	µ = 2.06 mm^−^^1^
α = 71.807 (3)°	*T* = 296 K
β = 86.612 (2)°	Block, yellow
γ = 80.709 (2)°	0.43 × 0.20 × 0.16 mm
*V* = 1622.59 (8) Å^3^	

### Data collection

**Table:**

Agilent SuperNova Dual (Cu at zero) Atlas diffractometer	6416 independent reflections
Radiation source: SuperNova (Cu) X-ray Source	5705 reflections with *I* > 2σ(*I*)
mirror	*R*_int_ = 0.031
Detector resolution: 10.4051 pixels mm^-1^	θ_max_ = 73.5°, θ_min_ = 3.0°
ω scans	*h* = −6→6
Absorption correction: multi-scan (*SADABS*; Sheldrick, 1996)	*k* = −19→19
*T*_min_ = 0.640, *T*_max_ = 0.720	*l* = −24→24
31859 measured reflections	

### Refinement

**Table:**

Refinement on *F*^2^	Secondary atom site location: difference Fourier map
Least-squares matrix: full	Hydrogen site location: inferred from neighbouring sites
*R*[*F*^2^ > 2σ(*F*^2^)] = 0.044	H atoms treated by a mixture of independent and constrained refinement
*wR*(*F*^2^) = 0.124	*w* = 1/[σ^2^(*F*_o_^2^) + (0.0609*P*)^2^ + 0.658*P*] where *P* = (*F*_o_^2^ + 2*F*_c_^2^)/3
*S* = 1.03	(Δ/σ)_max_ < 0.001
6416 reflections	Δρ_max_ = 0.46 e Å^−^^3^
379 parameters	Δρ_min_ = −0.32 e Å^−^^3^
9 restraints	Extinction correction: *SHELXL*, Fc^*^=kFc[1+0.001xFc^2^λ^3^/sin(2θ)]^-1/4^
Primary atom site location: structure-invariant direct methods	Extinction coefficient: 0.0031 (3)

### Special details

**Table:**

Geometry. All s.u.'s (except the s.u. in the dihedral angle between two l.s. planes) are estimated using the full covariance matrix. The cell s.u.'s are taken into account individually in the estimation of s.u.'s in distances, angles and torsion angles; correlations between s.u.'s in cell parameters are only used when they are defined by crystal symmetry. An approximate (isotropic) treatment of cell s.u.'s is used for estimating s.u.'s involving l.s. planes.
Refinement. Refinement of *F*^2^ against all reflections. The weighted *R*-factor *wR* and goodness of fit *S* are based on *F*^2^, conventional *R*-factors *R* are based on *F*, with *F* set to zero for negative *F*^2^. The threshold expression of *F*^2^ > σ(*F*^2^) is used only for calculating *R*-factors(gt) *etc*. and is not relevant to the choice of reflections for refinement. *R*-factors based on *F*^2^ are statistically about twice as large as those based on *F*, and *R*- factors based on all data will be even larger.

### Fractional atomic coordinates and isotropic or equivalent isotropic displacement parameters (Å^2^)

**Table:**

	*x*	*y*	*z*	*U*_iso_*/*U*_eq_	Occ. (<1)
Cl1	0.25688 (10)	1.14681 (4)	0.21378 (3)	0.05904 (16)	
O1	0.9124 (2)	0.88615 (9)	0.54464 (7)	0.0510 (3)	
N1	0.6002 (3)	0.87446 (10)	0.47672 (8)	0.0432 (3)	
N2	0.7802 (3)	0.99710 (11)	0.44015 (8)	0.0434 (3)	
H2	0.873 (4)	1.0339 (15)	0.4395 (11)	0.049 (6)*	
C1	0.4881 (3)	0.92923 (12)	0.41404 (9)	0.0409 (4)	
C2	0.6050 (3)	1.00686 (12)	0.39072 (9)	0.0400 (4)	
C3	0.5395 (3)	1.07491 (12)	0.32944 (9)	0.0434 (4)	
H3	0.6185	1.1259	0.3135	0.052*	
C4	0.3486 (3)	1.06323 (13)	0.29259 (9)	0.0441 (4)	
C5	0.2297 (3)	0.98786 (14)	0.31460 (10)	0.0469 (4)	
H5	0.1026	0.9832	0.2881	0.056*	
C6	0.2996 (3)	0.91879 (13)	0.37635 (10)	0.0463 (4)	
H6	0.2220	0.8674	0.3917	0.056*	
C7	0.7793 (3)	0.91679 (13)	0.49246 (9)	0.0430 (4)	
C11	0.5279 (4)	0.79200 (14)	0.52610 (10)	0.0521 (5)	
H11A	0.3529	0.7936	0.5218	0.063*	
H11B	0.5576	0.7914	0.5734	0.063*	
C12	0.6626 (4)	0.70491 (14)	0.51532 (11)	0.0555 (5)	
H12A	0.8369	0.7087	0.5110	0.067*	
H12B	0.6391	0.6548	0.5570	0.067*	
C13	0.5848 (4)	0.68309 (13)	0.45206 (11)	0.0526 (5)	
H13A	0.6122	0.7321	0.4100	0.063*	
H13B	0.4102	0.6797	0.4557	0.063*	
C14	0.7238 (4)	0.59384 (14)	0.44517 (12)	0.0573 (5)	
H14A	0.8975	0.5986	0.4395	0.069*	
H14B	0.7032	0.5457	0.4884	0.069*	
C15	0.6427 (4)	0.56742 (13)	0.38457 (12)	0.0578 (5)	
H15A	0.6546	0.6168	0.3415	0.069*	
H15B	0.4716	0.5589	0.3916	0.069*	
C16	0.7941 (5)	0.48115 (16)	0.37656 (15)	0.0746 (7)	
H16A	0.9640	0.4908	0.3680	0.090*	
H16B	0.7876	0.4326	0.4205	0.090*	
C17	0.7129 (7)	0.4510 (2)	0.31899 (18)	0.0965 (10)	
H17A	0.7396	0.4950	0.2739	0.116*	0.746 (7)
H17B	0.5388	0.4469	0.3241	0.116*	0.746 (7)
H17C	0.6367	0.5062	0.2847	0.116*	0.254 (7)
H17D	0.5809	0.4166	0.3392	0.116*	0.254 (7)
C18	0.8622 (12)	0.3558 (3)	0.3220 (2)	0.118 (2)	0.746 (7)
H18A	1.0352	0.3620	0.3170	0.141*	0.746 (7)
H18B	0.8392	0.3142	0.3684	0.141*	0.746 (7)
C19	0.8116 (13)	0.3189 (4)	0.2768 (4)	0.167 (3)	0.746 (7)
H19A	0.9265	0.2645	0.2810	0.250*	0.746 (7)
H19B	0.8222	0.3606	0.2304	0.250*	0.746 (7)
H19C	0.6481	0.3040	0.2853	0.250*	0.746 (7)
C18'	0.869 (2)	0.3959 (10)	0.2756 (8)	0.095 (4)*	0.254 (7)
H18C	0.7580	0.3612	0.2632	0.115*	0.254 (7)
H18D	0.9086	0.4405	0.2321	0.115*	0.254 (7)
C19'	1.056 (2)	0.3440 (8)	0.2934 (7)	0.084 (4)*	0.254 (7)
H19D	1.1224	0.3233	0.2548	0.126*	0.254 (7)
H19E	1.0224	0.2929	0.3321	0.126*	0.254 (7)
H19F	1.1727	0.3744	0.3073	0.126*	0.254 (7)
Cl1A	1.08169 (10)	−0.26825 (3)	0.31097 (3)	0.06033 (16)	
O1A	1.2494 (2)	0.09656 (8)	−0.01986 (6)	0.0430 (3)	
N1A	0.9635 (2)	0.06117 (9)	0.07076 (7)	0.0368 (3)	
N2A	1.3088 (2)	−0.03410 (9)	0.07700 (7)	0.0367 (3)	
H2A	1.445 (4)	−0.0579 (13)	0.0637 (10)	0.042 (5)*	
C1A	0.9508 (3)	−0.01099 (11)	0.13176 (9)	0.0347 (3)	
C2A	1.1701 (3)	−0.07145 (11)	0.13562 (9)	0.0345 (3)	
C3A	1.2157 (3)	−0.15133 (11)	0.18992 (9)	0.0386 (4)	
H3A	1.3617	−0.1914	0.1927	0.046*	
C4A	1.0307 (3)	−0.16868 (12)	0.24037 (9)	0.0414 (4)	
C5A	0.8119 (3)	−0.11037 (13)	0.23750 (9)	0.0425 (4)	
H5A	0.6934	−0.1251	0.2724	0.051*	
C6A	0.7689 (3)	−0.02980 (12)	0.18245 (9)	0.0395 (4)	
H6A	0.6228	0.0102	0.1798	0.047*	
C7A	1.1823 (3)	0.04634 (11)	0.03651 (9)	0.0358 (3)	
C11A	0.7749 (3)	0.13966 (11)	0.04375 (9)	0.0394 (4)	
H11C	0.6153	0.1195	0.0505	0.047*	
H11D	0.8009	0.1657	−0.0063	0.047*	
C12A	0.7736 (3)	0.21293 (11)	0.07887 (10)	0.0403 (4)	
H12C	0.9238	0.2394	0.0667	0.048*	
H12D	0.7690	0.1857	0.1294	0.048*	
C13A	0.5537 (3)	0.28753 (11)	0.05638 (10)	0.0416 (4)	
H13C	0.4042	0.2603	0.0661	0.050*	
H13D	0.5634	0.3166	0.0061	0.050*	
C14A	0.5394 (3)	0.35946 (12)	0.09357 (11)	0.0477 (4)	
H14C	0.5532	0.3293	0.1437	0.057*	
H14D	0.6785	0.3920	0.0788	0.057*	
C15A	0.3046 (3)	0.42779 (12)	0.07968 (11)	0.0486 (4)	
H15C	0.1654	0.3954	0.0950	0.058*	
H15D	0.2899	0.4577	0.0295	0.058*	
C16A	0.2936 (4)	0.49973 (13)	0.11649 (12)	0.0521 (5)	
H16C	0.4253	0.5352	0.0984	0.062*	
H16D	0.3220	0.4696	0.1662	0.062*	
C17A	0.0511 (4)	0.56397 (13)	0.10765 (12)	0.0513 (5)	
H17E	0.0253	0.5955	0.0581	0.062*	
H17F	−0.0811	0.5283	0.1244	0.062*	
C18A	0.0374 (4)	0.63392 (15)	0.14628 (14)	0.0618 (6)	
H18E	0.0751	0.6027	0.1953	0.074*	
H18F	0.1612	0.6728	0.1271	0.074*	
C19A	−0.2113 (4)	0.69262 (15)	0.14139 (15)	0.0666 (6)	
H19G	−0.2089	0.7353	0.1668	0.100*	
H19H	−0.3346	0.6548	0.1613	0.100*	
H19I	−0.2483	0.7250	0.0930	0.100*	

### Atomic displacement parameters (Å^2^)

**Table:**

	*U*^11^	*U*^22^	*U*^33^	*U*^12^	*U*^13^	*U*^23^
Cl1	0.0582 (3)	0.0627 (3)	0.0485 (3)	0.0098 (2)	−0.0121 (2)	−0.0134 (2)
O1	0.0467 (7)	0.0585 (8)	0.0468 (7)	−0.0017 (6)	−0.0114 (6)	−0.0160 (6)
N1	0.0388 (8)	0.0488 (8)	0.0421 (8)	−0.0026 (6)	−0.0022 (6)	−0.0155 (6)
N2	0.0385 (8)	0.0489 (8)	0.0446 (8)	−0.0044 (7)	−0.0046 (6)	−0.0173 (7)
C1	0.0345 (8)	0.0476 (9)	0.0414 (9)	0.0018 (7)	0.0004 (7)	−0.0191 (7)
C2	0.0326 (8)	0.0486 (9)	0.0417 (9)	0.0009 (7)	−0.0006 (7)	−0.0216 (7)
C3	0.0403 (9)	0.0458 (9)	0.0444 (9)	0.0003 (7)	0.0009 (7)	−0.0180 (8)
C4	0.0389 (9)	0.0520 (10)	0.0409 (9)	0.0072 (7)	−0.0030 (7)	−0.0199 (8)
C5	0.0350 (9)	0.0607 (11)	0.0495 (10)	0.0022 (8)	−0.0059 (7)	−0.0269 (9)
C6	0.0378 (9)	0.0531 (10)	0.0513 (10)	−0.0031 (8)	−0.0009 (8)	−0.0225 (8)
C7	0.0361 (9)	0.0513 (10)	0.0429 (9)	0.0024 (7)	−0.0026 (7)	−0.0202 (8)
C11	0.0491 (11)	0.0587 (11)	0.0450 (10)	−0.0084 (9)	0.0010 (8)	−0.0111 (9)
C12	0.0517 (11)	0.0519 (11)	0.0547 (11)	−0.0041 (9)	−0.0080 (9)	−0.0050 (9)
C13	0.0498 (11)	0.0475 (10)	0.0527 (11)	−0.0016 (8)	−0.0024 (9)	−0.0068 (8)
C14	0.0552 (12)	0.0441 (10)	0.0616 (12)	−0.0007 (9)	−0.0009 (10)	−0.0037 (9)
C15	0.0588 (12)	0.0414 (10)	0.0664 (13)	−0.0022 (9)	0.0010 (10)	−0.0098 (9)
C16	0.0811 (17)	0.0474 (12)	0.0872 (17)	0.0025 (11)	0.0077 (14)	−0.0166 (12)
C17	0.119 (3)	0.0678 (17)	0.111 (2)	−0.0117 (16)	0.022 (2)	−0.0447 (17)
C18	0.199 (6)	0.065 (2)	0.074 (3)	0.010 (3)	0.041 (3)	−0.023 (2)
C19	0.153 (6)	0.113 (5)	0.272 (9)	−0.012 (4)	0.016 (6)	−0.119 (6)
Cl1A	0.0607 (3)	0.0530 (3)	0.0560 (3)	−0.0081 (2)	0.0013 (2)	−0.0012 (2)
O1A	0.0374 (6)	0.0425 (6)	0.0453 (7)	−0.0005 (5)	0.0027 (5)	−0.0113 (5)
N1A	0.0302 (7)	0.0375 (7)	0.0436 (7)	0.0013 (5)	−0.0006 (6)	−0.0170 (6)
N2A	0.0275 (7)	0.0388 (7)	0.0432 (8)	0.0019 (5)	0.0023 (6)	−0.0156 (6)
C1A	0.0293 (8)	0.0379 (8)	0.0411 (8)	−0.0019 (6)	−0.0028 (6)	−0.0193 (7)
C2A	0.0279 (7)	0.0385 (8)	0.0415 (8)	−0.0033 (6)	−0.0005 (6)	−0.0195 (7)
C3A	0.0311 (8)	0.0373 (8)	0.0486 (9)	−0.0009 (6)	−0.0024 (7)	−0.0169 (7)
C4A	0.0403 (9)	0.0410 (9)	0.0438 (9)	−0.0081 (7)	−0.0021 (7)	−0.0131 (7)
C5A	0.0347 (9)	0.0524 (10)	0.0443 (9)	−0.0101 (7)	0.0051 (7)	−0.0196 (8)
C6A	0.0277 (8)	0.0484 (9)	0.0462 (9)	−0.0004 (7)	0.0000 (7)	−0.0229 (8)
C7A	0.0307 (8)	0.0373 (8)	0.0428 (9)	−0.0025 (6)	−0.0016 (7)	−0.0180 (7)
C11A	0.0311 (8)	0.0411 (9)	0.0470 (9)	0.0034 (6)	−0.0060 (7)	−0.0182 (7)
C12A	0.0327 (8)	0.0385 (8)	0.0512 (10)	0.0006 (6)	−0.0060 (7)	−0.0180 (7)
C13A	0.0365 (9)	0.0385 (9)	0.0480 (10)	0.0032 (7)	−0.0047 (7)	−0.0146 (7)
C14A	0.0437 (10)	0.0408 (9)	0.0598 (11)	0.0029 (7)	−0.0064 (8)	−0.0206 (8)
C15A	0.0428 (10)	0.0432 (9)	0.0617 (12)	0.0037 (8)	−0.0047 (8)	−0.0229 (9)
C16A	0.0442 (10)	0.0466 (10)	0.0686 (13)	0.0046 (8)	−0.0058 (9)	−0.0270 (9)
C17A	0.0435 (10)	0.0449 (10)	0.0684 (13)	0.0037 (8)	−0.0053 (9)	−0.0260 (9)
C18A	0.0461 (11)	0.0578 (12)	0.0918 (16)	0.0046 (9)	−0.0058 (10)	−0.0428 (12)
C19A	0.0532 (12)	0.0552 (12)	0.1005 (18)	0.0067 (9)	−0.0053 (12)	−0.0434 (13)

### Geometric parameters (Å, °)

**Table:**

Cl1—C4	1.7478 (19)	C18'—H18D	0.9700
O1—C7	1.236 (2)	C19'—H19D	0.9600
N1—C7	1.376 (2)	C19'—H19E	0.9600
N1—C1	1.395 (2)	C19'—H19F	0.9600
N1—C11	1.458 (2)	Cl1A—C4A	1.7435 (18)
N2—C7	1.363 (2)	O1A—C7A	1.234 (2)
N2—C2	1.382 (2)	N1A—C7A	1.376 (2)
N2—H2	0.83 (2)	N1A—C1A	1.389 (2)
C1—C6	1.379 (3)	N1A—C11A	1.457 (2)
C1—C2	1.405 (3)	N2A—C7A	1.370 (2)
C2—C3	1.373 (3)	N2A—C2A	1.385 (2)
C3—C4	1.388 (3)	N2A—H2A	0.85 (2)
C3—H3	0.9300	C1A—C6A	1.383 (2)
C4—C5	1.381 (3)	C1A—C2A	1.400 (2)
C5—C6	1.392 (3)	C2A—C3A	1.376 (2)
C5—H5	0.9300	C3A—C4A	1.391 (2)
C6—H6	0.9300	C3A—H3A	0.9300
C11—C12	1.515 (3)	C4A—C5A	1.383 (2)
C11—H11A	0.9700	C5A—C6A	1.389 (3)
C11—H11B	0.9700	C5A—H5A	0.9300
C12—C13	1.514 (3)	C6A—H6A	0.9300
C12—H12A	0.9700	C11A—C12A	1.521 (2)
C12—H12B	0.9700	C11A—H11C	0.9700
C13—C14	1.523 (3)	C11A—H11D	0.9700
C13—H13A	0.9700	C12A—C13A	1.522 (2)
C13—H13B	0.9700	C12A—H12C	0.9700
C14—C15	1.512 (3)	C12A—H12D	0.9700
C14—H14A	0.9700	C13A—C14A	1.523 (2)
C14—H14B	0.9700	C13A—H13C	0.9700
C15—C16	1.517 (3)	C13A—H13D	0.9700
C15—H15A	0.9700	C14A—C15A	1.519 (2)
C15—H15B	0.9700	C14A—H14C	0.9700
C16—C17	1.492 (4)	C14A—H14D	0.9700
C16—H16A	0.9700	C15A—C16A	1.519 (3)
C16—H16B	0.9700	C15A—H15C	0.9700
C17—C18'	1.543 (11)	C15A—H15D	0.9700
C17—C18	1.566 (5)	C16A—C17A	1.521 (2)
C17—H17A	0.9700	C16A—H16C	0.9700
C17—H17B	0.9700	C16A—H16D	0.9700
C17—H17C	0.9700	C17A—C18A	1.517 (3)
C17—H17D	0.9700	C17A—H17E	0.9700
C18—C19	1.282 (7)	C17A—H17F	0.9700
C18—H18A	0.9700	C18A—C19A	1.512 (3)
C18—H18B	0.9700	C18A—H18E	0.9700
C19—H19A	0.9600	C18A—H18F	0.9700
C19—H19B	0.9600	C19A—H19G	0.9600
C19—H19C	0.9600	C19A—H19H	0.9600
C18'—C19'	1.202 (12)	C19A—H19I	0.9600
C18'—H18C	0.9700		
			
C7—N1—C1	109.15 (15)	H19B—C19—H19C	109.5
C7—N1—C11	121.78 (16)	C19'—C18'—C17	126.9 (12)
C1—N1—C11	128.50 (16)	C19'—C18'—H18C	105.6
C7—N2—C2	110.23 (16)	C17—C18'—H18C	105.6
C7—N2—H2	123.3 (15)	C19'—C18'—H18D	105.6
C2—N2—H2	126.5 (15)	C17—C18'—H18D	105.6
C6—C1—N1	132.18 (18)	H18C—C18'—H18D	106.1
C6—C1—C2	121.04 (17)	C18'—C19'—H19D	109.5
N1—C1—C2	106.78 (15)	C18'—C19'—H19E	109.5
C3—C2—N2	131.50 (17)	H19D—C19'—H19E	109.5
C3—C2—C1	121.83 (16)	C18'—C19'—H19F	109.5
N2—C2—C1	106.67 (16)	H19D—C19'—H19F	109.5
C2—C3—C4	116.14 (17)	H19E—C19'—H19F	109.5
C2—C3—H3	121.9	C7A—N1A—C1A	109.46 (13)
C4—C3—H3	121.9	C7A—N1A—C11A	123.64 (14)
C5—C4—C3	123.14 (17)	C1A—N1A—C11A	126.86 (13)
C5—C4—Cl1	118.22 (14)	C7A—N2A—C2A	109.97 (13)
C3—C4—Cl1	118.63 (15)	C7A—N2A—H2A	122.0 (13)
C4—C5—C6	120.23 (17)	C2A—N2A—H2A	127.7 (13)
C4—C5—H5	119.9	C6A—C1A—N1A	131.97 (15)
C6—C5—H5	119.9	C6A—C1A—C2A	121.06 (15)
C1—C6—C5	117.62 (18)	N1A—C1A—C2A	106.96 (13)
C1—C6—H6	121.2	C3A—C2A—N2A	131.38 (14)
C5—C6—H6	121.2	C3A—C2A—C1A	121.85 (15)
O1—C7—N2	127.51 (18)	N2A—C2A—C1A	106.77 (14)
O1—C7—N1	125.34 (18)	C2A—C3A—C4A	116.09 (15)
N2—C7—N1	107.16 (15)	C2A—C3A—H3A	122.0
N1—C11—C12	113.94 (16)	C4A—C3A—H3A	122.0
N1—C11—H11A	108.8	C5A—C4A—C3A	123.16 (16)
C12—C11—H11A	108.8	C5A—C4A—Cl1A	118.98 (14)
N1—C11—H11B	108.8	C3A—C4A—Cl1A	117.85 (13)
C12—C11—H11B	108.8	C4A—C5A—C6A	120.05 (16)
H11A—C11—H11B	107.7	C4A—C5A—H5A	120.0
C13—C12—C11	115.51 (17)	C6A—C5A—H5A	120.0
C13—C12—H12A	108.4	C1A—C6A—C5A	117.78 (15)
C11—C12—H12A	108.4	C1A—C6A—H6A	121.1
C13—C12—H12B	108.4	C5A—C6A—H6A	121.1
C11—C12—H12B	108.4	O1A—C7A—N2A	127.19 (15)
H12A—C12—H12B	107.5	O1A—C7A—N1A	125.98 (15)
C12—C13—C14	112.76 (17)	N2A—C7A—N1A	106.83 (14)
C12—C13—H13A	109.0	N1A—C11A—C12A	113.23 (14)
C14—C13—H13A	109.0	N1A—C11A—H11C	108.9
C12—C13—H13B	109.0	C12A—C11A—H11C	108.9
C14—C13—H13B	109.0	N1A—C11A—H11D	108.9
H13A—C13—H13B	107.8	C12A—C11A—H11D	108.9
C15—C14—C13	114.34 (17)	H11C—C11A—H11D	107.7
C15—C14—H14A	108.7	C11A—C12A—C13A	111.52 (14)
C13—C14—H14A	108.7	C11A—C12A—H12C	109.3
C15—C14—H14B	108.7	C13A—C12A—H12C	109.3
C13—C14—H14B	108.7	C11A—C12A—H12D	109.3
H14A—C14—H14B	107.6	C13A—C12A—H12D	109.3
C14—C15—C16	113.1 (2)	H12C—C12A—H12D	108.0
C14—C15—H15A	109.0	C12A—C13A—C14A	112.85 (15)
C16—C15—H15A	109.0	C12A—C13A—H13C	109.0
C14—C15—H15B	109.0	C14A—C13A—H13C	109.0
C16—C15—H15B	109.0	C12A—C13A—H13D	109.0
H15A—C15—H15B	107.8	C14A—C13A—H13D	109.0
C17—C16—C15	114.6 (2)	H13C—C13A—H13D	107.8
C17—C16—H16A	108.6	C15A—C14A—C13A	114.10 (16)
C15—C16—H16A	108.6	C15A—C14A—H14C	108.7
C17—C16—H16B	108.6	C13A—C14A—H14C	108.7
C15—C16—H16B	108.6	C15A—C14A—H14D	108.7
H16A—C16—H16B	107.6	C13A—C14A—H14D	108.7
C16—C17—C18'	128.3 (6)	H14C—C14A—H14D	107.6
C16—C17—C18	109.5 (3)	C14A—C15A—C16A	113.61 (16)
C18'—C17—C18	35.5 (5)	C14A—C15A—H15C	108.8
C16—C17—H17A	109.8	C16A—C15A—H15C	108.8
C18'—C17—H17A	74.6	C14A—C15A—H15D	108.8
C18—C17—H17A	109.8	C16A—C15A—H15D	108.8
C16—C17—H17B	109.8	H15C—C15A—H15D	107.7
C18'—C17—H17B	117.5	C15A—C16A—C17A	114.03 (16)
C18—C17—H17B	109.8	C15A—C16A—H16C	108.7
H17A—C17—H17B	108.2	C17A—C16A—H16C	108.7
C16—C17—H17C	105.2	C15A—C16A—H16D	108.7
C18'—C17—H17C	105.2	C17A—C16A—H16D	108.7
C18—C17—H17C	139.6	H16C—C16A—H16D	107.6
H17A—C17—H17C	36.9	C18A—C17A—C16A	114.01 (17)
H17B—C17—H17C	76.1	C18A—C17A—H17E	108.8
C16—C17—H17D	105.2	C16A—C17A—H17E	108.8
C18'—C17—H17D	105.2	C18A—C17A—H17F	108.8
C18—C17—H17D	84.3	C16A—C17A—H17F	108.8
H17A—C17—H17D	134.3	H17E—C17A—H17F	107.6
H17B—C17—H17D	30.1	C19A—C18A—C17A	113.28 (18)
H17C—C17—H17D	105.9	C19A—C18A—H18E	108.9
C19—C18—C17	117.6 (5)	C17A—C18A—H18E	108.9
C19—C18—H18A	107.9	C19A—C18A—H18F	108.9
C17—C18—H18A	107.9	C17A—C18A—H18F	108.9
C19—C18—H18B	107.9	H18E—C18A—H18F	107.7
C17—C18—H18B	107.9	C18A—C19A—H19G	109.5
H18A—C18—H18B	107.2	C18A—C19A—H19H	109.5
C18—C19—H19A	109.5	H19G—C19A—H19H	109.5
C18—C19—H19B	109.5	C18A—C19A—H19I	109.5
H19A—C19—H19B	109.5	H19G—C19A—H19I	109.5
C18—C19—H19C	109.5	H19H—C19A—H19I	109.5
H19A—C19—H19C	109.5		
			
C7—N1—C1—C6	−179.22 (18)	C16—C17—C18'—C19'	26 (2)
C11—N1—C1—C6	−7.9 (3)	C18—C17—C18'—C19'	−42.3 (13)
C7—N1—C1—C2	0.62 (18)	C7A—N1A—C1A—C6A	−177.64 (17)
C11—N1—C1—C2	171.91 (16)	C11A—N1A—C1A—C6A	−0.1 (3)
C7—N2—C2—C3	−179.52 (17)	C7A—N1A—C1A—C2A	0.82 (18)
C7—N2—C2—C1	0.68 (18)	C11A—N1A—C1A—C2A	178.39 (14)
C6—C1—C2—C3	−0.7 (3)	C7A—N2A—C2A—C3A	178.36 (17)
N1—C1—C2—C3	179.40 (14)	C7A—N2A—C2A—C1A	−0.95 (18)
C6—C1—C2—N2	179.08 (15)	C6A—C1A—C2A—C3A	−0.6 (2)
N1—C1—C2—N2	−0.78 (18)	N1A—C1A—C2A—C3A	−179.32 (14)
N2—C2—C3—C4	−178.71 (17)	C6A—C1A—C2A—N2A	178.74 (15)
C1—C2—C3—C4	1.1 (2)	N1A—C1A—C2A—N2A	0.07 (17)
C2—C3—C4—C5	−0.7 (2)	N2A—C2A—C3A—C4A	−178.81 (16)
C2—C3—C4—Cl1	−179.55 (12)	C1A—C2A—C3A—C4A	0.4 (2)
C3—C4—C5—C6	−0.1 (3)	C2A—C3A—C4A—C5A	0.0 (3)
Cl1—C4—C5—C6	178.81 (13)	C2A—C3A—C4A—Cl1A	−179.05 (12)
N1—C1—C6—C5	179.77 (17)	C3A—C4A—C5A—C6A	−0.3 (3)
C2—C1—C6—C5	0.0 (2)	Cl1A—C4A—C5A—C6A	178.81 (13)
C4—C5—C6—C1	0.4 (3)	N1A—C1A—C6A—C5A	178.69 (16)
C2—N2—C7—O1	179.57 (17)	C2A—C1A—C6A—C5A	0.4 (2)
C2—N2—C7—N1	−0.30 (19)	C4A—C5A—C6A—C1A	0.0 (3)
C1—N1—C7—O1	179.92 (16)	C2A—N2A—C7A—O1A	−178.60 (16)
C11—N1—C7—O1	7.9 (3)	C2A—N2A—C7A—N1A	1.45 (18)
C1—N1—C7—N2	−0.20 (18)	C1A—N1A—C7A—O1A	178.65 (15)
C11—N1—C7—N2	−172.19 (15)	C11A—N1A—C7A—O1A	1.0 (3)
C7—N1—C11—C12	−93.8 (2)	C1A—N1A—C7A—N2A	−1.40 (18)
C1—N1—C11—C12	95.8 (2)	C11A—N1A—C7A—N2A	−179.06 (14)
N1—C11—C12—C13	−73.8 (2)	C7A—N1A—C11A—C12A	−101.69 (18)
C11—C12—C13—C14	−178.88 (17)	C1A—N1A—C11A—C12A	81.1 (2)
C12—C13—C14—C15	177.21 (18)	N1A—C11A—C12A—C13A	−172.36 (14)
C13—C14—C15—C16	176.70 (19)	C11A—C12A—C13A—C14A	176.96 (15)
C14—C15—C16—C17	177.7 (2)	C12A—C13A—C14A—C15A	−172.27 (16)
C15—C16—C17—C18'	151.9 (8)	C13A—C14A—C15A—C16A	−179.44 (17)
C15—C16—C17—C18	−173.2 (3)	C14A—C15A—C16A—C17A	−175.42 (18)
C16—C17—C18—C19	178.6 (6)	C15A—C16A—C17A—C18A	178.20 (19)
C18'—C17—C18—C19	−51.9 (10)	C16A—C17A—C18A—C19A	−175.7 (2)

### Hydrogen-bond geometry (Å, °)

**Table:**

*D*—H···*A*	*D*—H	H···*A*	*D*···*A*	*D*—H···*A*
N2—H2···O1^i^	0.83 (2)	1.96 (2)	2.778 (2)	170 (2)
N2A—H2A···O1A^ii^	0.85 (2)	1.95 (2)	2.7937 (18)	171.1 (19)

Symmetry codes: (i) −*x*+2, −*y*+2, −*z*+1; (ii) −*x*+3, −*y*, −*z*.
